# Type I interferons during host–fungus interactions: Is antifungal immunity going viral?

**DOI:** 10.1371/journal.ppat.1010740

**Published:** 2022-08-25

**Authors:** Marina Pekmezovic, Axel Dietschmann, Mark S. Gresnigt

**Affiliations:** Junior Research Group Adaptive Pathogenicity Strategies, Leibniz Institute for Natural Product Research and Infection Biology-Hans-Knoell Institute, Jena, Germany; University of Maryland, Baltimore, UNITED STATES

## 1. Type I interferons (IFN-I): Beyond the viral control

**Fig 1:**
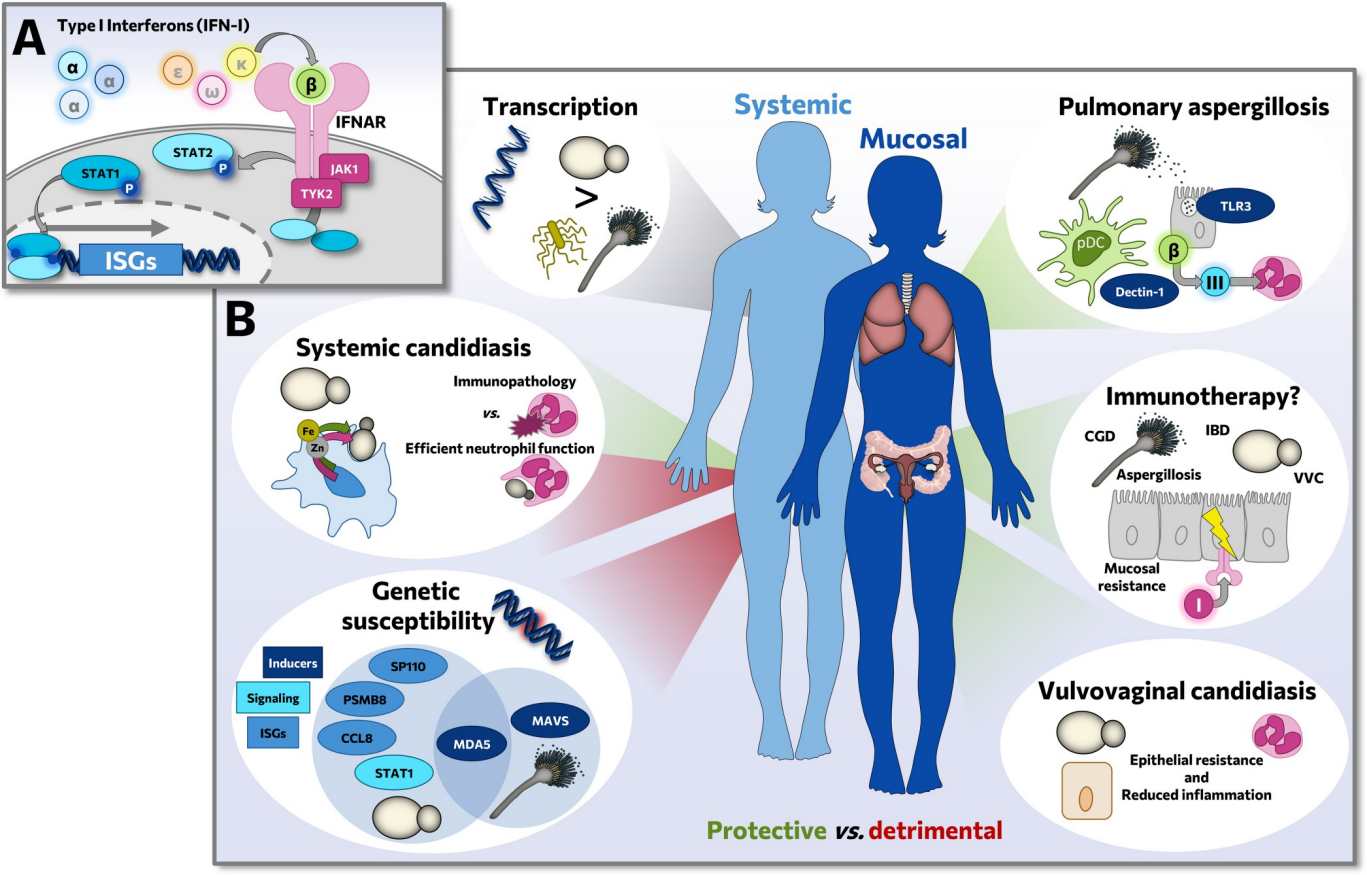
(A) Overview of mechanistic IFN-I signaling, activating ISG expression via route of the IFNAR heterodimer and downstream interaction of the kinases JAK1 and TYK2 with STAT1/2 transcription factors. (B) Overview of how IFN-I steer immune responses in infections caused by *Candida* species and *A*. *fumigatus*. *C*. *albicans* causes specific enrichment of IFN-I responses, and genetic variations in the IFN-I pathway are linked to susceptibility to candidiasis and aspergillosis. While remaining controversial in systemic candidiasis, IFN-I are largely found protective on mucosae and during aspergillosis rather a protective role is observed. This renders IFN-I lucrative as potential immunotherapeutics on mucosal surfaces. CCL8, C-C motif chemokine ligand 8/monocyte chemoattractant protein 2; CGD, Chronic granulomatous disease; IBD, inflammatory bowel disease; IFN-I/I, type I interferon; III, type III interferon; IFNAR, IFN-α/β receptor; ISG, interferon-stimulated gene; JAK1, Janus kinase 1; MAVS, mitochondrial antiviral signaling protein; MDA5, melanoma differentiation-associated protein 5/interferon induced with helicase C domain 1; pDC, plasmacytoid dendritic cell; PSMB8, proteasome 20S subunit beta-8; SP110, speckled 110 kD/interferon-induced protein 41; STAT, signal transducer and activator of transcription; TLR, Toll-like receptor; TYK2, tyrosine kinase 2; VVC, vulvovaginal candidiasis.

Type I interferons (IFN-I) are crucial in antiviral host defense. However, increasing evidence suggests that IFN-I also play important roles during infections with nonviral pathogens. IFN-I, a family of multiple cytokines that all activate the IFN-α/β receptor (IFNAR), induce expression of interferon-stimulated genes (ISGs) via JAK-STAT1/2 signaling (Fig 1A). ISG-encoded proteins are involved in different processes, many going beyond antiviral defense. In contrast to their well-understood role during viral infections, IFN-I exert both beneficial and detrimental effects to the host. IFN-I orchestrate host defense through antimicrobial and immunomodulatory properties, but also instigate tissue damage and strong proinflammatory responses [1]. While this contrasting role is incompletely understood, a growing number of studies implicated IFN-I responses during fungal infections caused by *Candida* species and *Aspergillus fumigatus* (Fig 1B).

## 2. IFN-I during invasive fungal infections: A controversial role?

Multiple systemic candidiasis models show activation of IFN-I responses, but whether this mediates protection remains unclear. Mice lacking IFNAR showed increased susceptibility to candidiasis [2], suggesting a protective role of IFN-I. This attenuation of *Ifnar1*^−/−^ mice correlated with reduced neutrophil recruitment [1]. Contrastingly, reduced neutrophil recruitment resulting from *Ifnar1* deficiency was also observed to improve survival by limiting immunopathology [3]. Indirect detrimental effects of IFN-I were observed during *Candida parapsilosis* infection through IL-27 induced by IFNAR signaling [4]. IL-27R-deficient mice showed a progressive reduction in *C*. *parapsilosis* burden, suggesting that IL-27 compromises fungal clearance. Yet, no changes in survival were observed [4]. Finally, *Ifnar1*^−/−^ mice showed lower *Candida glabrata* burdens 7 days postinfection [5], proposing that IFN-I promotes its persistence, although these differences were no longer observed at later stages [5].

Several studies hint toward an explanation for the detrimental effect of IFN-I through interference with specific antifungal defense processes. IFN-β, for example, promoted pathology and death from candidiasis by inducing an ISG with tetratricopeptide repeats 2 (IFIT2), which suppressed production of NADPH-oxidase-dependent reactive oxygen species [6]. During *C*. *glabrata* infection, IFN-I were reported to dysregulate macrophage metal homeostasis, which exemplary favored fungal iron acquisition [7].

While these studies partially solve the puzzle of IFN-I during systemic candidiasis, we have to be mindful that effects of IFN-I may differ between murine models and humans. Polymorphisms in IFN-I-related genes associate with an increased susceptibility to candidemia [8], hinting toward their importance in humans. Missense variants in *IFIH1* encoding the IFI-I-inducing receptor MDA5 increased susceptibility to candidiasis [9]. Additionally, IFN-I responses were specifically enriched in human PBMCs upon the interaction with *C*. *albicans*, not only in comparison to bacteria [8], but also to *Rhizopus oryzae* and *A*. *fumigatus* [10].

Nevertheless, IFN-I also seems to play an important role in resistance against aspergillosis. The MDA5/MAVS receptor system mediates IFN-I and type III IFN (IFN-III) responses and resistance to aspergillosis [11], and the same polymorphisms in *IFIH1* that mediate susceptibility to candidiasis also predispose for aspergillosis [12]. TLR3, another nucleic acid sensor implicated in defense against aspergillosis [13], initiates the IFN-β pathway upon recognition of double-stranded *A*. *fumigatus* RNA by epithelial cells [14]. IFN-I produced upon pulmonary *A*. *fumigatus* infection was found to regulate neutrophil antifungal potential via IFN-III signaling [15]. Secretion of IFN-I also was found dependent on dectin-1-mediated recognition of *A*. *fumigatus* β-glucan [16]. Plasmacytoid dendritic cells (pDCs), which are important during antiviral defense, were identified as a potential driver of IFN-I responses in *A*. *fumigatus* infection, and both pDCs and IFNAR seem crucial for resistance [17].

## 3. IFN-I on mucosal surfaces: A potential role in fungal commensalism?

On mucosal surfaces, epithelial cells are key players in shaping innate immunity—toward tolerance in case of commensal microbes or toward the initiation of host defense upon infection. Several studies demonstrated that IFN-I modulate epithelial responses, based on their variety of biological effects and being both pro- and anti-inflammatory. Intestinal cells may rely on IFN-I signaling to guide the immune system to differentiate between commensals and pathogens [18]. Specific probiotic formulations were observed to increase local IFN-α release, modifying the resident gut flora, which inhibited *Candida* growth [19]. It seems IFN-I can promote commensalism via at least 2 mechanisms—acting directly on host cells to keep inflammation under control [18] and by shaping the intestinal microbiota composition, thereby indirectly controlling potential opportunistic pathogens, such as *Candida* spp. [19].

On the vaginal mucosa, IFN-I signaling and stimulation of ISGs was a common signature of early vaginal epithelial cell responses to infection with different *Candida* species [20]. Later during the infection, the IFN-I response was not enriched, suggesting a predominant role during early infection, when there is no epithelial damage and the interaction between host and fungus resembles commensalism. This is supported by the observation that healthy women have higher IFN-β levels in vaginal fluid compared to women with vulvovaginal candidiasis (VVC) [21]. Interestingly, the opposite was observed for IFN-α, which was elevated in VVC patients [21]. Protective effects of IFN-I were shown both in vivo and in vitro models of VVC [20,22].

Compared to systemic candidiasis, where the role of IFN-I is rather unclear, effects during mucosal infections suggest a beneficial role by increasing epithelial resistance. Whether IFN-I-induced epithelial resistance can be exploited for increased resistance to infection remains to be explored.

## 4. Fungi and viruses: Cross-protection or increased susceptibility by IFN-I?

Fungal infections are increasingly observed in association with viral infections. This raises the question whether the IFN-I pathway plays a role in pathogenesis of these virus-associated fungal infections. *Aspergillus* species can cause superinfections in patients critically ill from viral disease, even when otherwise immunocompetent [23]. Devastating examples are influenza associated and recently also COVID-associated pulmonary aspergillosis, IAPA or CAPA, respectively. Both diseases substantially induce IFN-I responses, and in severe COVID, this exacerbates immunopathology [24]. Contrastingly, severe COVID also was associated with development of autoantibodies against protective IFN-I [25]. Given the role of IFN-I signaling in restricting aspergillosis [11,12,16,17], an exhaustive negative feedback of the IFN-I response may partially explain CAPA predisposition in severe COVID cases [15]. Comparative studies of influenza or COVID patients with patients developing IAPA or CAPA may shed light on the role of IFN-I in their pathogenesis.

In the context of IAPA, influenza-induced STAT1, but not STAT2, signaling was found to compromise neutrophil recruitment and increasing susceptibility to aspergillosis [26].

Thinking of viral–fungal comorbidities, fungal infections can be superinfections following viral disease. However, this order of events rather is an exception as our immune system is constantly exposed to ubiquitous and commensal fungi. Exemplary, in fungal respiratory allergy with subsequent viral infection, different mechanisms are observed. IFN-I does not always abrogate, but may even amplify type 2 immunity and eosinophilic inflammation [27]. As *A*. *fumigatus*-activated eosinophils can protect against infection with respiratory viruses [28,29], this pathway could represent a mechanism, how fungal priming of IFN-I responses builds antiviral countermeasures. Given the potential of commensal *Candida* species to induce IFN-I responses [8,10,20], it may be worth investigating, whether their colonization shapes mucosal antiviral immunity.

## 5. Is IFN-I immunotherapy an option?

Immunotherapy is an increasingly recognized essential strategy to improve the outcome of fungal infections. These therapies either can augment a compromised immune system or suppress detrimental inflammatory responses. Thus, this is particularly attractive for infections in immunocompromised patients or infections associated with immunopathology.

The capacity of IFN-I to improve several aspects of antifungal host defense warrants their exploration as candidates for immunotherapy of mucosal fungal infections.

Particularly, treatment of VVC is complex due to the interplay between fungal pathogenicity and immunopathology underlying its pathogenesis. While immunotherapy for VVC has not yet been broadly explored, IFNα was successful in increasing resistance to VVC in a rat model [22]. Besides, IFN-I treatment can have diverse beneficial effects during VVC by increasing epithelial resistance to infection [20,22] and inhibiting detrimental inflammatory responses [20]. This evidence suggesting a protective role of IFN-I at mucosal barriers supports that specifically mucosal, rather than systemic, disease may benefit from such therapy. Concerning aspergillosis, chronic granulomatous disease (CGD) patients are another group that potentially could ultimately benefit from IFN-I immunotherapy, as IFN-I-inducing poly I:C treatment in mice neutrophil-dependent improved outcome of aspergillosis [30]. Furthermore, exogenous IFN-I administration rescued inadequate antifungal responses in *dectin-1*^*−/−*^ mice [16]. IFN-I signaling is well documented for its role in maintaining intestinal barrier homeostasis. Therefore, it is tempting to speculate that IFN-I immunotherapy may be a valuable approach to reduce *C*. *albicans* translocation through improving epithelial barrier function. It is, however, difficult to estimate how such an immunotherapy affects immunocompromised patients. Yet in the context of inflammatory bowel diseases (IBDs), IFN-I are discussed as an immunomodulatory treatment strategy. Given the evidence for a key role of *C*. *albicans* pathogenicity mechanisms in IBD [31], IFN-I therapy may both modulate inflammatory responses and increase epithelial resistance to fungal pathogenicity. IFN-I mediate cross-talk between epithelial cells and the immune system during commensalism and infection, potentially making them an attractive therapeutic target for fungal infections in the gut, lung, and vaginal mucosa.
